# A Systematic Review of the Use of mHealth in Oral Health Education among Older Adults

**DOI:** 10.3390/dj11080189

**Published:** 2023-08-08

**Authors:** Reinhard Chun Wang Chau, Khaing Myat Thu, Akhilanand Chaurasia, Richard Tai Chiu Hsung, Walter Yu-Hang Lam

**Affiliations:** 1 Faculty of Dentistry, The University of Hong Kong, Hong Kong 999077, China; rcwchau@hku.hk (R.C.W.C.); khaing@hku.hk (K.M.T.); 2 Faculty of Dental Sciences, King George’s Medical University, Lucknow 226003, India; chaurasiaakhilanand49@gmail.com; 3 Department of Computer Science, Hong Kong Chu Hai College, Hong Kong 999077, China; richardhsung@chuhai.edu.hk

**Keywords:** telemedicine, aged, oral health, health education, dental

## Abstract

Oral diseases are largely preventable. However, as the number of older adults is expected to increase, along with the high cost and various barriers to seeking continuous professional care, a sustainable approach is needed to assist older adults in maintaining their oral health. Mobile health (mHealth) technologies may facilitate oral disease prevention and management through oral health education. This review aims to provide an overview of existing evidence on using mHealth to promote oral health through education among older adults. A literature search was performed across five electronic databases. A total of five studies were identified, which provided low to moderate evidence to support using mHealth among older adults. The selected studies showed that mHealth could improve oral health management, oral health behavior, and oral health knowledge among older adults. However, more quality studies regarding using mHealth technologies in oral health management, oral health behavior, and oral health knowledge among older adults are needed.

## 1. Introduction

According to the United Nations (UN), the number of persons aged 65 years and over is expected to double by 2050 [1]. Individuals’ oral health tends to decline as their age increases, and such decline has been linked to various systemic diseases [2,3,4]. Therefore, as the number of older adults increases, appropriate actions would be needed to maintain their oral health [5].

With advancements in oral healthcare over the past few decades, it is observed that the number of older adults maintaining natural teeth for their lifetime has increased [6]. However, natural teeth are susceptible to many dental diseases, such as caries, periodontal disease, and tooth wear, thus leading to increased demand for dental treatments.

It is recognized that many oral diseases are mostly behavioral problems and are, therefore, preventable with appropriate approaches [7,8]. Effective self-care oral hygiene measures, such as toothbrushing and interdental cleaning, are essential to oral disease prevention and control. In addition, studies have revealed that older adults who receive regular behavioral instructions tend to have a lower prevalence of caries, gingival inflammation, or tooth loss than their counterparts who rely solely on continuous professional attention [7,9,10,11]. However, given the limited physical functioning of many older adults, their ability to seek professional care is limited. Moreover, it may not be sustainable to send teams of dentists to visit older adults frequently [12,13].

Due to recent technology developments, adopting mobile computing and communication technologies in healthcare and public health is now a possible solution. Mobile health or mHealth is defined by the World Health Organization (WHO) as “the use of mobile devices (mobile phones, patient monitoring devices, and personal digital assistants) for medical and public health practice” [14]. In general health areas, clinicians have been testing using mobile electronic devices (MEDs), like wearable sensors, to provide older adults with real-time feedback and personalized recommendations for disease management, as well as to promote long-term behavior change with the aid of guided behavioral approaches [15,16,17]. The application of MEDs may allow dentists to provide remote clinical instructions and support oral health behavior change [18,19,20].

The WHO oral health program, headed by Benoit Varenne, has been working on leveraging technology to improve oral health outcomes and promote universal oral health coverage through areas such as oral health promotion, disease prevention, and integrated care [21]. There have been studies investigating the adoption of teledentistry, especially mHealth, as clinical tools for promoting oral health and oral health management for patients who could not visit dental facilities [22,23]. In addition, several reviews have looked at the evidence to support the use of mHealth technologies as oral health knowledge interventions for the general population [22,24] and children [25]. However, there seems to be a lack of summary of evidence regarding using mHealth technologies as oral health knowledge interventions among older adults, and past reviews provided mixed acceptance of mHealth among older adults regarding other health aspects [26,27].

The objectives of this review were to (1) identify the uses of mobile computing and communication technologies by dental professionals in the context of oral health management, oral health behavior, and oral health knowledge of older adults; (2) assess the effectiveness of mHealth regarding different aspects related to oral health, including oral health management, oral health behavior, and oral health knowledge, among older adults; and (3) evaluate the acceptability of mHealth among older adults in oral health management, oral health behavior, and oral health knowledge.

## 2. Materials and Methods

This review followed the Preferred Reporting Items for Systematic Reviews and Meta-analyses (PRISMA) statement [28].

### 2.1. Review Question and Criteria

This review adopted the Population, Intervention, Control, and Outcomes (PICO) framework to answer the question “Is the application of mHealth tools effective in promoting oral health among older adults?” The population (P) of this review was older adults aged 60 years and older [29], without other restrictions. The intervention (I) was any non-clinical oral health intervention, targeting oral health management, oral health behavior, or oral health knowledge, with MEDs. The comparator (C) was conventional (oral health) education (CE) on oral health management, oral health behavior, and oral health knowledge [30,31]. The outcomes (Os) considered in this review were any improvement in terms of oral health management, oral health behavior, or oral health knowledge observed from the cohorts, with or without follow-up assessments [32,33].

Both randomized and quasi-randomized control trials were included. Studies without statistical analysis and studies that did not use mHealth technologies were excluded. Studies that focused on the perspectives of healthcare workers instead of patients were also excluded. The detailed selection criteria are listed in Table 1.

### 2.2. Search Strategy

A literature search was performed across 4 electronic databases, including PubMed, MEDLINE, Scopus, and Web of Science, with no filters applied to include the maximum number of studies. An additional search was performed via Google Scholar from inception until February 2023. The search strategy applied was as follows:

“[(Aged) OR (elderly)) OR (old adults)) OR (senior citizen)] AND [(Dental Care for aged) OR (oral health)) OR (dental health)) OR (oral hygiene)) OR (dental health education)) OR (oral health promotion)) OR (oral health education)) OR (oral hygiene instruction)] AND [(telemedicine) OR (teledentistry)) OR (mHealth)) OR (eHealth)) OR (mobile application)) OR (telecommunication)) OR (m-Oral Health)) OR (e-Oral Health)]”.

### 2.3. Study Selection

Studies were checked for duplications, and title and abstract screening was performed independently by 2 researchers (R.C.W.C., K.M.T.) using an online platform (Covidence, Australia) [34]. Disagreements were solved by discussion. Full-text reading was then performed to select eligible articles by the abovementioned 2 researchers independently using the same online platform. Disagreements were again solved by discussion.

### 2.4. Data Extraction

Data extraction was conducted independently by the abovementioned 2 researchers using the mentioned online platform, and assessment of the risk of bias was performed independently by the 2 researchers using the National Institutes of Health (NIH) study quality assessment tools [35,36]. The following study features were extracted: (i) method of delivery [37], (ii) content delivered [37], (iii) length per session of the intervention [38], (iv) clinical outcome(s) [37,38], (v) participant-reported outcome(s) [39], (vi) qualitative usability and acceptability [40], and (vii) oral health knowledge outcome(s) [41]. The data extracted were assessed by a third researcher (W.Y.-H.L.) to ensure data quality. In addition, any study’s correspondence author(s) with details missing from the publication were contacted.

## 3. Results

### 3.1. Search Results

**Figure 1:**
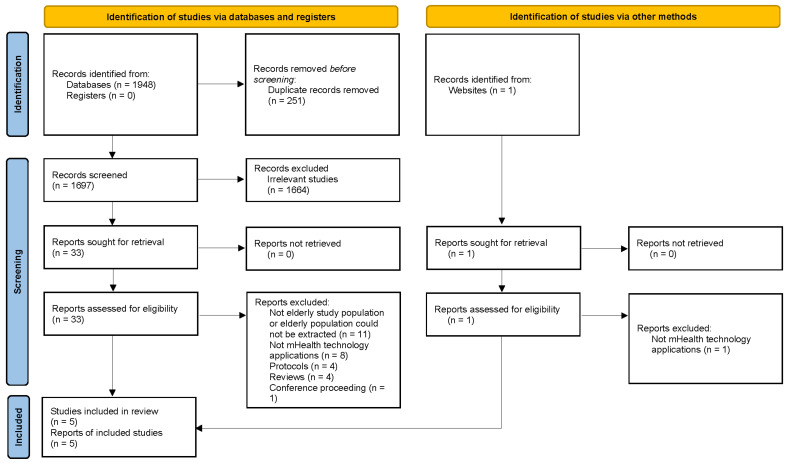
PRISMA flowchart of this review.

A total of 1949 studies were retrieved through the primary literature search. After removing duplicates, 1698 studies were screened, and of those, 33 studies were shortlisted for full-text assessment based on screening of their titles and abstracts. Next, the full text of the shortlisted studies was assessed for eligibility, and finally, five studies were selected for this review (Figure 1).

### 3.2. Study Characteristics

A total of 422 participants were included in the selected studies (n = 5), where 268 were assigned to receive mHealth interventions (**Interventions**), and 154 were assigned to control groups (**Controls**). The mean number of participants per study was 84.4, the median was 75, and the number of participants ranged from 46 to 150.

All five selected studies delivered the mHealth interventions through smartphones [42,43,44,45,46], with three of them adopting smartphone applications (APPs) [42,43,44], while one utilized web-based interventions accessible using smartphone web browsers [45], and one delivered interventions through the short message service (SMS) [46]. The participants of two studies were recruited among older adults functioning independently in local communities [44,45], while two recruited through social welfare services [42,43], and one recruited from a dental clinic [46] (Table 2).

### 3.3. Format and Content Delivery

Four studies adopted audio–visual materials to deliver oral health education messages [42,43,44,45], while one chose text-only delivery through SMS [46]. Four selected studies adopted various interactive features when designing the oral health education materials [42,43,44,45], while one was a one-way delivery of educational materials [46].

All five studies covered toothbrushing instructions in their oral health education materials. Three studies provided the participants with information regarding common oral diseases among older adults [42,45,46], while two briefly covered all oral diseases [43,44]. Instructions on proper denture maintenance was covered in four out of the five studies [43,44,45,46]. Oral motor exercises and mouth massage were included in one study [43]. All studies provided evidence-based knowledge with references (Table 3).

### 3.4. Outcomes

The five included studies reported five key outcomes, including (i) clinical, (ii) participant-reported, (iii) qualitative, and (iv) oral health knowledge outcomes, as well as (v) acceptability of mHealth interventions (Table 4).

#### 3.4.1. Clinical Outcomes

Only two studies reported clinical outcomes [42,43]. One study reported no significant difference across three clinical indices (the O’Leary Index, tongue coating, and Löe and Silness Index) between the intervention and control groups [42]. The other study reported significant improvement in the Plaque Index; however, no significant differences were reported for the number of functional teeth and tongue coating [43].

#### 3.4.2. Participant-Reported Outcomes

Significant decrease in self-reported oral dryness and a significant increase in self-reported swallowing-related quality of life (SWAL-QoL) and tongue pressure after the intervention were reported in one study [43]. In another study, participants reported a significant increase in willingness to use dental floss after intervention [46].

#### 3.4.3. Qualitative Outcomes

Two studies reported that the mHealth intervention positively impacted oral health behavior and improved oral health knowledge among participants [45,46]. One study reported that participants wanted more than non-individualized oral health education [45].

#### 3.4.4. Oral Health Knowledge Outcomes

Significant improvements in oral health behavior or oral health knowledge after intervention were reported in three studies [42,44,45]. There was one study that reported a significant increase in knowledge about preventing dental caries and periodontal diseases [45].

#### 3.4.5. Acceptability of mHealth Intervention(s)

Two studies reported the acceptability of mHealth as an intervention for oral health management, oral health behavior, and oral health knowledge [45,46]. One study reported that the older adult participants “showed strong support” and “valued” the mHealth intervention, as the participants recognized “the importance of communicating dental information through an online approach”; the participants were also reported to enjoy, feel comfortable, and feel respected with the mHealth intervention as older learners [45]. Another study reported a significantly higher acceptance of mHealth intervention compared to CE; 89% of intervention participants would recommend the mHealth intervention to others, compared to 68% in the control group (*p* < 0.05) [46].

### 3.5. Assessment of Risk of Bias

The summary of the assessment of the risk of bias in the selected studies is reported in Table 5. None of the five studies was considered “good” (low risk of bias). Two [44,45,46] were considered “poor” (high risk of bias), while three [42,43] were considered “fair” (unclear risk of bias) [35].

For [42], the intervention allocation was not concealed, and the participants and the assessors were not blinded. The dropout rate was higher than 15%. All these factors resulted in “fair”.

For [43], the intervention allocation was not concealed, and the participants and the assessors were not blinded. The dropout rate was higher than 15%. The information on participant adherence to interventions was not provided. All these factors resulted in “fair”.

For [44], the assessors were not blinded, and participants were changed in the mid-course of the study. The study also failed to adopt an interrupted time-series design, and it was impossible to determine if the statistical analysis was appropriate. Thus, all these factors resulted in “poor”.

For [45], the participants’ selection criteria were not clearly defined, and non-eligible participants were recruited. The assessors were not blinded, and the study failed to adopt an interrupted time-series design. It was not possible to determine if the statistical analysis was appropriate. In addition, the dropout rate was more than 30% (33%), and according to the NIH quality assessment guidelines, it should be considered a “fatal flaw”. Thus, the study was rated as “poor”.

For [46], the intervention allocation was not concealed, and the participants and the assessors were not blinded. The information on participant adherence to interventions was not provided. The information on whether a validated and reliable measurement was used consistently throughout the study was also not reported. In addition, the dropout rate was more than 30% (55%), and according to the NIH quality assessment guidelines, it should be considered a “fatal flaw”. Thus, the study was rated as “poor”.

## 4. Discussion

This study reviewed the evidence that supports the use of mHealth technologies, such as MEDs, to perform oral health education among older adults. The existing evidence suggested that efforts were being made by dental professionals to use mobile computing and communication technologies to facilitate the oral health management, oral health behavior, and oral health knowledge of older adults. The acceptability was high, though the reported effectiveness was mixed. The result of this review aligned with other studies regarding mHealth in other aspects of healthcare in terms of benefits from mHealth interventions and the potential for better disease management [38,40]. However, the quality of the evidence was not strong. Most studies reported short and few interactions between the participants and the respective research team, suggesting that the outcomes observed, both clinical and behavioral, may be due to other factors, such as participants’ self-care using the provided mHealth tools.

This review widened the scope of existing studies regarding using mHealth technologies to facilitate oral health education, with special emphasis on older adults [22,24,25]. Any form of oral health education intervention with mHealth was included in this study, and all were accessible with MEDs. Among all five studies, a certain form of reinforcement of oral health behavior and oral health knowledge was observed, which suggested that mHealth, with stronger interactions between the participants and the clinicians, could be an ideal tool for repetition and reinforcement, aligning with outcomes of previous studies on oral health behavior and oral health knowledge [22,60].

The willingness to use mHealth technologies among the older adult participants was also observed, suggesting an increase in digital literacy among the older adults as reported in other studies (even though their skills might be limited) [61], which might facilitate the autonomy of older adults and encourage self-efficacy [24].

However, the studies included in this review have several limitations. Most studies did not include baseline clinical examinations, and there was a lack of long-term follow-up, so the potential impacts of mHealth on the oral health of older adults were still not investigated. There was also a lack of information about participants’ adherence to the full intervention and the recruitment rate. Moreover, most of the included studies reported high dropout rates. These factors impaired the quality of the study outcomes.

There were also other limitations to this review. While the effects and acceptance of mHealth technologies were observed, there was limited information to explain such a phenomenon, thus limiting the possibility of exploring each variable and its roles in the outcomes. In addition, given the complex and non-comparable nature of study designs and measurement, meta-analysis was not possible, which limited the quantitative assessment of the impacts of mHealth technologies on older adults. Furthermore, the studies included in this review had limited generalizability, as they were conducted in only four countries (South Korea, Egypt, Australia, and the United Kingdom). This represents a small portion of the various healthcare systems available worldwide. Therefore, readers should exercise caution when interpreting the results of this study.

Future studies investigating the use of mHealth should consider facilitators and barriers of mHealth and older adults, like oral health education materials that are easy to follow and can capture participants’ attention. In addition, investigations of the impact of mHealth on the oral health education of older adults should be paired with adequate clinical assessments, as well as longitudinal follow-ups, to examine short-term and long-term impacts. To improve assessments of the impacts of oral health education interventions, it is recommended to report the baseline information, including the oral health status, the behavioral characteristics of participants, and their oral health knowledge. The dental conditions of the participants such as the degree of edentulism, as well as their prosthetic status, should be reported in future studies.

Advancements in mobile computing and communication technologies have enabled a more personalized approach to oral health management, oral health behavior, and oral health knowledge. One included study reported negative comments from the participants toward non-individualized information [46], which indicated that older adults might require individualized oral health information to feel motivated to adopt mHealth oral health knowledge. Recent studies have demonstrated the use of photographs for site specific gum disease detection [62,63]. Therefore, oral health education tailored to individual needs can be delivered. It is anticipated that improvements in smartphone cameras may facilitate the detection and monitoring of oral diseases, allowing early identification among high-risk patient groups and precise management. As newer-generation mobile networks continue to improve, remote consultation and oral health education via teledentistry [64] may also become more accessible, as well as the use of cloud-based AI for providing more personalized oral health management [65].

## 5. Conclusions

The existing evidence suggests that mHealth is being used by dental professionals to improve oral health management, oral health behavior, and oral health knowledge among older adults with high acceptability and mixed effectiveness. Such technology may potentially become a valuable tool for promoting oral health. However, the quality of the available studies was fair to poor. More quality studies regarding using mHealth technologies to facilitate oral health management, oral health behavior, and oral health knowledge among older adults are needed.

## Figures and Tables

**Table 1:** Inclusion/exclusion criteria of this review.

Inclusion Criteria	Exclusion Criteria
- Original clinical studies. - Studies on oral health education or oral hygiene instruction utilizing mHealth technologies. - Studies published in English. - Studies that performed statistical analysis.	- Non-original clinical studies (commentaries, reviews, protocols, etc.). - Conference proceedings. - Studies focused on healthcare workers instead of patients.

**Table 2:** The characteristics of included studies.

Author (Year), Country	Participants ^1^	Interventions and Controls	Outcome(s) Measured
Lee (2023), South Korea [42]	Enrolled in a senior welfare center. 65 to 85+ years old. (Gender of recruited participants not specified.) N = 90/73. Intervention: n = 25. Control 1: n = 22. Control 2: n = 26.	**Intervention** Access to oral health education APP for 5 weeks, with 2 reminders per week. **Control 1** 30 min oral health education lecture for 5 weeks, twice a week. 15 min practice after each lecture. **Control 2** No oral health education.	- Oral health knowledge. ^+^ - Oral health perception. ^+^ - Oral Health Impact Profile-14 (OHIP-14). ^^^ - General Oral Health Assessment Index (GOHAI). ^^^ - O’Leary Index. ^^^ - Tongue Coating Index. ^^^ - Löe and Silness Index. ^^^
Ki (2021), South Korea [43]	Enrolled in a social service program. 65 to 75+ years old. (Gender of recruited participants not specified.) N = 46/40. Intervention: n = 20. Control: n = 20.	**Intervention** Access to oral health education APP for 6 weeks, with an unspecified number of 1-to-1 customized education sessions of up to 50 min. **Control** No oral health education.	- Oral health behavior. ^+^ - Number of functional teeth. ^^^ - Plaque Index. ^^^ - Tongue Coating Index. ^^^ - Oral frailty. ^+^ - OHIP-14. ^^^ - GOHAI. ^^^ - Dietary factors. ^+^
Khalil (2020), Egypt [44]	Independent older adults in the community. 60 to 70+ years old. 26 males, 41 females. N = 67/67. Intervention: n = 67. No external control.	**Intervention** Access to oral health education through WhatsApp for 4 weeks, with 2 sessions per week and each lasting no more than 15 min. **Control** Baseline oral health status of the participants.	- Oral health knowledge. ^+^ - Oral health perception. ^+^ - Geriatric Self-Efficacy Scale for Oral Health (GSEOH). ^+^
Marino (2016), Australia [45]	Independent older adults in the community. (Age of recruited participants not specified.) (Gender of recruited participants not specified.) N = 75/47. Intervention: n = 47. No external control.	**Intervention** 10 oral health education modules were provided on a website over 10 weeks, 1 per week. Additional sessions were provided for a catch-up. Each session lasted from 27 to 38 min. **Control** Baseline oral health status of the participants.	- Oral health knowledge. ^+^ - Oral health perception. ^+^ - Self-defined self-efficacy score. ^+^
Wanyonyi (2022), the United Kingdom [46]	Attendees of a dental clinic. 71.7 years old (mean). 85 male, 65 female. N = 150/68. Intervention: n = 40. Control: n = 28.	**Intervention** Three oral health education text messages per week for 10 weeks. **Control** Oral health education leaflets were delivered at the dental clinic.	- Perceived helpfulness of the program. - Willingness to recommend the program to others. - OHIP-14. ^^^ - 12-Item General Health Questionnaire (GHQ-12). ^+^ - Unspecified clinical assessments. ^^^

^1^ Participant characteristics, age, gender, number of participants recruited/completed the studies, and number of participants per group; ^^^ Outcomes related to oral health management; ^+^ Outcomes related to oral health behavior or oral health knowledge.

**Table 3:** Format and content delivery of selected studies in this review.

Author (Year)	Format of Delivery (mHealth Technology), Length per Session (If Applicable)	Content Delivered	Reference(s)
Lee (2023) [42]	Audio–visual materials (mobile APP on smartphones).	- Oral health problems in old adulthood: dental caries and gingival disease; dry mouth and bad breath. - Oral management: toothbrushing and denture management; diet and smoking cessation; dental scaling and periodic oral check-ups. - Oral health education video on toothbrushing. - Interactive quizzes and workbooks.	[47]
Ki (2021) [43]	Audio–visual materials (mobile APP on smartphones), 50 min per session.	- Trot songs (a genre of Korean popular music) [48] adapted with oral health education script. - Oral exercise education consisting of oral gum exercises and tongue exercises. - Intraoral and extraoral massage. - Customized oral hygiene intervention, including brushing and denture care methods. - Self-care of oral health. - Interactive workbooks.	[49,50,51]
Khalil (2020) [44]	Audio–visual materials (mobile APP on smartphones), 15 min per session.	- Importance of oral health and its indicators. - Basic components of the oral cavity and age-related changes in the oral cavity. - Risk factors for oral health problems in older adults. - Gingivitis: causes, manifestations, and management. - Tooth decay: causes, stages, complications, and how to prevent it. - Halitosis: causes and management. - Dry mouth: causes, manifestations, and management. - Tooth sensitivity; causes, manifestations, and management. - Tooth brux: causes, manifestations, and management. - Dental neuritis: causes, manifestations, and management. - First aid for tooth fractures. - Mouth ulcer: causes, manifestations, and management. - Oral cancer: manifestations. - Steps of toothbrushing, care for a toothbrush, and tooth flossing. - Components of healthy food to maintain oral health. - How to care for dentures. - Guidelines to prevent oral health problems in older adults, steps of self-examination of the oral cavity. - Interactive WhatsApp groups.	[52,53,54,55,56]
Marino (2016) [45]	Audio–visual materials (web-based and accessible on smartphones or computers), 27 to 38 min per session.	- Oral health and aging. - Dental caries. - Periodontal disease. - Oral cancer. - What to do with remaining teeth. - Care of dentures. - Dry mouth (xerostomia). - Oral health and nutrition. - Use of oral healthcare services. - Oral health and general health. - Interactive quizzes.	[57,58]
Wanyonyi (2022) [46]	Text-only materials (SMS on smartphones).	- Toothbrushing behaviors. - Flossing. - Fluoride and mouth rinse to use. - Denture cleaning. - Dry mouth.	[59]

**Table 4:** Summary of key outcomes that were reported in included studies.

Author (Year)	Clinical Outcome(s)	Participant-Reported Outcome(s)	Qualitative Outcome(s)	Oral Health Knowledge Outcome(s)	Acceptability
Lee (2023) [42]	No significant improvement: - O’Leary Index. - Tongue coating. - Löe and Silness Index.	Not reported.	Not reported.	Significant improvement in oral health knowledge.	Not reported.
Ki (2021) [43]	Significant improvement: - Plaque Index. No significant improvement: - Number of functional teeth. - Tongue coating.	Significant improvement: - Oral dryness. - Swallowing-related quality of life (SWAL-QoL). - Tongue pressure.	Not reported.	Not reported.	Not reported.
Khalil (2020) [44]	Not reported	Not reported	Not reported.	Significant improvement in oral health literacy.	Not reported.
Marino (2016) [45]	Not reported	Not reported.	- Improved oral health awareness. - Improved oral health behaviors. - Improved oral health perceptions. - Participants were unsatisfied with non-individualized materials.	Significant improvement in oral health knowledge.	- Strong participant support. - Positive feedback on mHealth interventions.
Wanyonyi (2022) [46]	Not reported.	Significant improvement: - Willingness to use dental floss.	- Improved oral health awareness. - Improved oral health behaviors. - Improved oral health perceptions.	Not reported.	- High acceptance (89%) reported.

**Table 5:** Summary of risk of bias in the selected studies.

Author (Year)	Type of NIH Quality Assessment Tool (Detailed Assessment)	Quality Rating (Score)
Lee (2023) [42]	Controlled Intervention Studies (Table A1).	Fair (10/14)
Ki (2021) [43]	Controlled Intervention Studies (Table A2).	Fair (9/14)
Khalil (2020) [44]	Before–After (Pre–Post) Studies With No Control Group (Table A3).	Poor (8/12)
Marino (2016) [45]	Before–After (Pre–Post) Studies With No Control Group (Table A4)	Poor (6/12) ^1^
Wanyonyi (2022) [46]	Controlled Intervention Studies (Table A5)	Poor (8/14) ^1^

^1^ Fatal flaw(s) [35] were observed in the study.

## Data Availability

No new data were created or analyzed in this study. Data sharing is not applicable to this article.

## Appendix A

**Table A1:** Assessment of risk of bias for Lee et al. (2023) [42].

Criteria	Yes	No	Other (CD, NR, NA) *
1. Was the study described as randomized, a randomized trial, a randomized clinical trial, or an RCT?	✓		
2. Was the method of randomization adequate (i.e., use of randomly generated assignment)?	✓		
3. Was the treatment allocation concealed (so that assignments could not be predicted)?		✓	
4. Were study participants and providers blinded to treatment group assignment?		✓	
5. Were the people assessing the outcomes blinded to the participants’ group assignments?		✓	
6. Were the groups similar at baseline on important characteristics that could affect outcomes (e.g., demographics, risk factors, co-morbid conditions)?	✓		
7. Was the overall dropout rate from the study at endpoint 20% or lower of the number allocated to treatment?	✓		
8. Was the differential dropout rate (between treatment groups) at endpoint 15 percentage points or lower?		✓	
9. Was there high adherence to the intervention protocols for each treatment group?	✓		
10. Were other interventions avoided or similar in the groups (e.g., similar background treatments)?	✓		
11. Were outcomes assessed using valid and reliable measures, implemented consistently across all study participants?	✓		
12. Did the authors report that the sample size was sufficiently large to be able to detect a difference in the main outcome between groups with at least 80% power?	✓		
13. Were the outcomes reported or sub-groups analyzed pre-specified (i.e., identified before analyses were conducted)?	✓		
14. Were all randomized participants analyzed in the group to which they were originally assigned, i.e., did they use an intention-to-treat analysis?	✓		

* CD, cannot determine; NA, not applicable; NR, not reported.

**Table A2:** Assessment of risk of bias for Ki et al. (2021) [43].

Criteria	Yes	No	Other (CD, NR, NA) *
1. Was the study described as randomized, a randomized trial, a randomized clinical trial, or an RCT?	✓		
2. Was the method of randomization adequate (i.e., use of randomly generated assignment)?	✓		
3. Was the treatment allocation concealed (so that assignments could not be predicted)?		✓	
4. Were study participants and providers blinded to treatment group assignment?		✓	
5. Were the people assessing the outcomes blinded to the participants’ group assignments?		✓	
6. Were the groups similar at baseline on important characteristics that could affect outcomes (e.g., demographics, risk factors, co-morbid conditions)?	✓		
7. Was the overall dropout rate from the study at endpoint 20% or lower of the number allocated to treatment?	✓		
8. Was the differential dropout rate (between treatment groups) at endpoint 15 percentage points or lower?		✓	
9. Was there high adherence to the intervention protocols for each treatment group?			CD
10. Were other interventions avoided or similar in the groups (e.g., similar background treatments)?	✓		
11. Were outcomes assessed using valid and reliable measures, implemented consistently across all study participants?	✓		
12. Did the authors report that the sample size was sufficiently large to be able to detect a difference in the main outcome between groups with at least 80% power?	✓		
13. Were the outcomes reported or sub-groups analyzed pre-specified (i.e., identified before analyses were conducted)?	✓		
14. Were all randomized participants analyzed in the group to which they were originally assigned, i.e., did they use an intention-to-treat analysis?	✓		

* CD, cannot determine; NA, not applicable; NR, not reported.

**Table A3:** Assessment of risk of bias for Khalil et al. (2020) [44].

Criteria	Yes	No	Other (CD, NR, NA) *
1. Was the study question or objective clearly stated?	✓		
2. Were eligibility/selection criteria for the study population pre-specified and clearly described?	✓		
3. Were the participants in the study representative of those who would be eligible for the test/service/intervention in the general or clinical population of interest?	✓		
4. Were all eligible participants that met the pre-specified entry criteria enrolled?	✓		
5. Was the sample size sufficiently large to provide confidence in the findings?	✓		
6. Was the test/service/intervention clearly described and delivered consistently across the study population?	✓		
7. Were the outcome measures pre-specified, clearly defined, valid, reliable, and assessed consistently across all study participants?	✓		
8. Were the people assessing the outcomes blinded to the participants’ exposures/interventions?		✓	
9. Was the loss to follow-up after baseline 20% or less? Were those lost to follow-up accounted for in the analysis?	✓ ^+^		
10. Did the statistical methods examine changes in outcome measures from before to after the intervention? Were statistical tests performed that provided p-values for the pre-to-post changes?	✓		
11. Were outcome measures of interest taken multiple times before the intervention and multiple times after the intervention (i.e., did they use an interrupted time-series design)?		✓	
12. If the intervention was conducted at a group level (e.g., a whole hospital, a community, etc.), did the statistical analysis take into account the use of individual-level data to determine effects at the group level?			CD
Reason(s) to be considered “Poor”	^+^ Changes in participants to keep the same sample size during the study		

* CD, cannot determine; NA, not applicable; NR, not reported.

**Table A4:** Assessment of risk of bias for Marino et al. (2016) [45].

Criteria	Yes	No	Other (CD, NR, NA) *
1. Was the study question or objective clearly stated?	✓		
2. Were eligibility/selection criteria for the study population pre-specified and clearly described?		✓	
3. Were the participants in the study representative of those who would be eligible for the test/service/intervention in the general or clinical population of interest?	✓		
4. Were all eligible participants that met the pre-specified entry criteria enrolled?		✓	
5. Was the sample size sufficiently large to provide confidence in the findings?	✓		
6. Was the test/service/intervention clearly described and delivered consistently across the study population?	✓		
7. Were the outcome measures pre-specified, clearly defined, valid, reliable, and assessed consistently across all study participants?	✓		
8. Were the people assessing the outcomes blinded to the participants’ exposures/interventions?		✓	
9. Was the loss to follow-up after baseline 20% or less? Were those lost to follow-up accounted for in the analysis?		✓ ^+^	
10. Did the statistical methods examine changes in outcome measures from before to after the intervention? Were statistical tests performed that provided p-values for the pre-to-post changes?	✓		
11. Were outcome measures of interest taken multiple times before the intervention and multiple times after the intervention (i.e., did they use an interrupted time-series design)?		✓	
12. If the intervention was conducted at a group level (e.g., a whole hospital, a community, etc.), did the statistical analysis take into account the use of individual-level data to determine effects at the group level?			NA
Reason(s) to be considered “Poor”	^+^ High dropout rate (33%), which was considered a fatal flaw according to guidelines [35]		

* CD, cannot determine; NA, not applicable; NR, not reported.

**Table A5:** Assessment of risk of bias for Wanyonyi et al. (2022) [46].

Criteria	Yes	No	Other (CD, NR, NA) *
1. Was the study described as randomized, a randomized trial, a randomized clinical trial, or an RCT?	✓		
2. Was the method of randomization adequate (i.e., use of randomly generated assignment)?	✓		
3. Was the treatment allocation concealed (so that assignments could not be predicted)?		✓	
4. Were study participants and providers blinded to treatment group assignment?		✓	
5. Were the people assessing the outcomes blinded to the participants’ group assignments?		✓	
6. Were the groups similar at baseline on important characteristics that could affect outcomes (e.g., demographics, risk factors, co-morbid conditions)?	✓		
7. Was the overall dropout rate from the study at endpoint 20% or lower of the number allocated to treatment?		✓ ^+^	
8. Was the differential dropout rate (between treatment groups) at endpoint 15 percentage points or lower?	✓		
9. Was there high adherence to the intervention protocols for each treatment group?			CD
10. Were other interventions avoided or similar in the groups (e.g., similar background treatments)?	✓		
11. Were outcomes assessed using valid and reliable measures, implemented consistently across all study participants?			NR
12. Did the authors report that the sample size was sufficiently large to be able to detect a difference in the main outcome between groups with at least 80% power?	✓		
13. Were the outcomes reported or sub-groups analyzed pre-specified (i.e., identified before analyses were conducted)?	✓		
14. Were all randomized participants analyzed in the group to which they were originally assigned, i.e., did they use an intention-to-treat analysis?	✓		
Reason(s) to be considered “Poor”	^+^ High dropout rate (55%), which was considered a fatal flaw according to guidelines [35]		

* CD, cannot determine; NA, not applicable; NR, not reported.
